# Digital Signal Processing Reveals Circadian Baseline Oscillation in Majority of Mammalian Genes

**DOI:** 10.1371/journal.pcbi.0030120

**Published:** 2007-06-15

**Authors:** Andrey A Ptitsyn, Sanjin Zvonic, Jeffrey M Gimble

**Affiliations:** 1 Department of Microbiology, Immunology and Pathology, College of Veterinary and Biomedical Sciences, Colorado State University, Fort Collins, Colorado, United States of America; 2 Stem Cell Laboratory, Clinical Nutrition Research Unit, Pennington Biomedical Research Center, Louisiana State University System, Baton Rouge, Louisiana, United States of America; 3 Cell Biology Core Facility, Clinical Nutrition Research Unit, Pennington Biomedical Research Center, Louisiana State University System, Baton Rouge, Louisiana, United States of America; Harvard University, United States of America

## Abstract

In mammals, circadian periodicity has been described for gene expression in the hypothalamus and multiple peripheral tissues. It is accepted that 10%–15% of all genes oscillate in a daily rhythm, regulated by an intrinsic molecular clock. Statistical analyses of periodicity are limited by the small size of datasets and high levels of stochastic noise. Here, we propose a new approach applying digital signal processing algorithms separately to each group of genes oscillating in the same phase. Combined with the statistical tests for periodicity, this method identifies circadian baseline oscillation in almost 100% of all expressed genes. Consequently, circadian oscillation in gene expression should be evaluated in any study related to biological pathways. Changes in gene expression caused by mutations or regulation of environmental factors (such as photic stimuli or feeding) should be considered in the context of changes in the amplitude and phase of genetic oscillations.

## Author Summary

Prior studies have reported that ~15% of expressed genes show a circadian expression pattern in association with a specific function. A series of experimental and computational studies of gene expression in various murine tissues has led us to a different conclusion. By applying a new analysis strategy and a number of alternative algorithms, we identify baseline oscillation in almost 100% of all genes. While the phase and amplitude of oscillation vary between different tissues, circadian oscillation remains a fundamental property of every gene. Reanalysis of previously published data also reveals a greater number of oscillating genes than was previously reported. This suggests that circadian oscillation is a universal property of all mammalian genes, although phase and amplitude of oscillation are tissue-specific and remain associated with a gene's function. We hypothesize that the cell's metabolic respiratory cycle drives the oscillatory pattern of gene expression. These findings imply that biological pathways should be considered as dynamic systems of genes oscillating in coordination with each other.

## Introduction

Periodic patterns are widespread in the behavior, physiology, and gene expression of almost all organisms from cyanobacteria to humans. The most prevalent oscillating pattern is circadian, or approximately daily rhythm. It is commonly accepted that up to 15% of all mammalian genes follow this rhythm entrained by photic stimuli resulting from the alternating light and dark periods of the day. The molecular clock driven by positive and negative transcriptional regulatory feedback loops has been extensively studied [1]. The central circadian clock is located in the suprachiasmic nucleus in the brain [2], but active molecular clocks, presumably synchronized by suprachiasmic nucleus–mediated activity, have also been reported in peripheral tissues. Many researchers have demonstrated that the analysis of circadian rhythms is important for a complete understanding of both physiology and pathology in mammalian and other species [3,4]. The idea of multiplicity and diversity of biological oscillators in a living cell has been championed in much earlier works of Selkov [5,6]. Robustness of a system with multiple oscillators has been also demonstrated in theoretical models [7]. Our recent findings demonstrate that the prominence and impact of oscillatory processes in living tissues may still be underestimated.

Analysis of time series gene expression data for periodic patterns presents a significant challenge. The number of time points in such studies is limited by the affordability of microarrays. Microarrays, widely used in analysis of gene expression, allow simultaneous observation of thousands of genes. However, microarray expression intensity of each specific gene may have a high degree of stochastic variation and are not always reliable for technological reasons. More precise qRT-PCR analysis can be done to validate the microarray expression estimation, but only for a small number of selected genes. In a typical circadian study, biological samples are taken every 2–4 h, and the duration of the study covers no more than two complete periods (48 h). Thus, the resulting profile for each gene is characterized by a very low sampling rate. Combined with a high level of stochastic noise, this makes the application of standard periodicity tests difficult due to insufficient statistical power. A number of algorithmic approaches have been applied recently to identify periodically expressed genes among thousands of time series profiles [8,9]. However, each of these methods only considers one gene expression profile at a time and lacks the statistical power to identify the circadian component in more than a small percentage of genes. We recognized and acknowledged this problem in our earlier publications [10,11]. Here, we propose a different approach to the analysis of periodicity in microarray gene expression profiles that gives an alternative, and possibly more realistic, estimation of the scale of oscillation in a living tissue.

## Methods

Each gene expression profile is preprocessed by z-score transformation, which equalizes the scale of variation between genes and centers each profile on zero. We start with phase classification, assigning each gene a phase based on the maximal correlation to an ideal cosine curve:

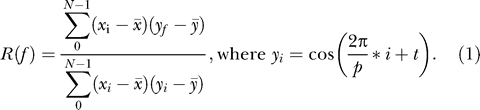



Each gene expression profile is correlated to a series of cosine curves generated with a phase shift *t* equal to the interval between sample collection points (4 h for most datasets). The phase *t_max_* that produces the highest correlation is considered the most likely phase for the gene *x*. This method is superior to the assignment of phase based on the position of peaks because it takes into account all data points in the series. A pattern of alternating up-and-down trends, detected by this approach, is more robust compared with the position of a peak, especially in short noisy time series, where a single uncertainty of two similar height peaks may result in a 4-h phase difference. We perform the phase assignment for all expression profiles before analyzing periodicity. The provisionally assigned phase does not imply that the expression profile is periodic. For each profile *x,* autocorrelation with the circadian lag (*R_c_*) is calculated:

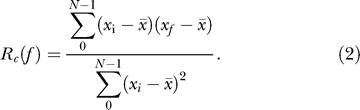



**Figure 1:**
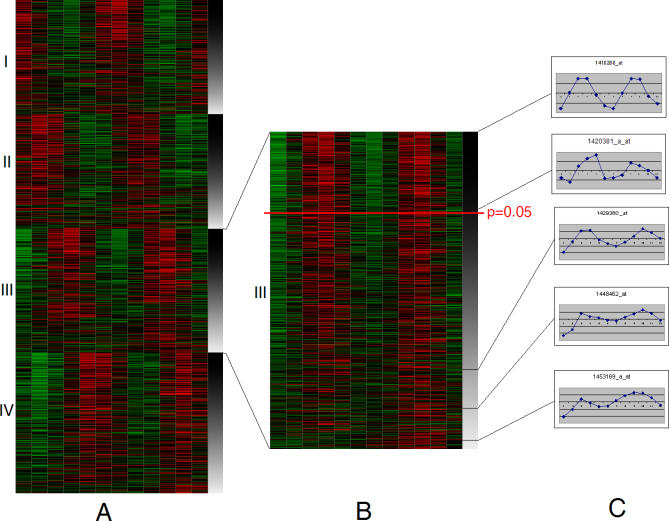
Analysis of Circadian Frequency in Separate Groups Classified by Phase The heatmap (A) presents all 22,689 genes, separated in four same-phase groups. In each phase group, all gene expression profiles are sorted in the descending order of the likeliness of periodicity (which can be estimated by a *p*-value of periodicity test or in this case by autocorrelation with shift by one circadian period; see Text S1). The circadian autocorrelation is depicted on the right margin by intensity of gray (black corresponds to 100% correlation, white to no correlation between two circadian periods in each profile). Each group of same-phase profiles can be considered separately (B). Only a small portion of genes pass the standard periodicity test (above red tick mark at *p* = 0.05, estimated by *Pt*-test). However, the pattern of two red and two green zones (elevated and reduced expression, correspondingly) extends beyond the cutoff. Periodic pattern is also obvious in many of the expression profiles that have failed the test. A few examples of such profiles (C) are taken from the top, above the *p*-value cutoff, and from the bottom of the same-phase class of profiles. These profiles, although not perfect, still demonstrate an obvious two-hump pattern over 2 d of observation.

All profiles are sorted first by phase and then by descending order of autocorrelation with the circadian lag *R_c_*. Previous studies [10] indicate that autocorrelation values with the circadian lag, if sorted, form a smooth line with no apparent separation between oscillating and nonoscillating fractions. From this point on in our analysis, each same-phase group of genes is studied separately from the other groups. By linking together all profiles of the same phase with equalized range of variation (amplitude) in order of descending circadian autocorrelation *R_c_*, we generate a continuous stream *C_ph_* of measurements. The stream carries a signal (i.e., circadian frequency), which is clear on one end and deteriorates into stochastic noise on the opposite end. Figure 1 outlines the process described above by plotting the real data derived from murine liver [11]. In this setting, the problem of identifying circadian oscillating genes can be formulated as finding a point in the continuum at which the circadian frequency deteriorates below recognition at the *p* = 0.05 confidence level. To solve this problem, we apply algorithms commonly used in digital signal processing. This set of algorithms and approaches is widely applied in various areas of electronics, acoustics, and medicine. Digital representation of analog signals allows the introduction of powerful filters, increasing signal to noise ratio. Since we operate on microarray data already converted into a digital form, this makes adaptation of digital signal processing algorithms developed for other engineering applications fast and effective.

Each same-phase continuum is treated with a low-pass frequency filter, and polynomial smoothing is applied. We analyze each phase fraction separately to detect the point at which the circadian signal deteriorates beyond the *p* = 0.05 significance cutoff. A window *W*, covering a few circadian periods of the stream, is transformed to frequency domain using discrete Fourier transform (DFT). The resulting periodogram *I_w_* is compared to a periodogram of a randomly permutated window *W_r_* using the Kolmogorov-Smirnov (K-S) goodness of fit test. We repeat the test, shifting the start of the window *W* from the beginning (i.e., most clearly oscillating genes) towards the end (most noisy profiles). The frame is shifted each time by the number of time points, making one complete gene expression profile. Once the point at which the *I_w_* does not differ significantly from a random periodogram *I_wr_* is detected, this is designated as the “cutoff”; we count all gene expression profiles above this point as having a circadian signal. The schematic overview of the algorithm of analysis of periodicity in phase continuum is given in Figure S1. By summing results from each phase group, we estimate the total number of circadian oscillating genes. The length of the window *W* is a parameter that defines the level of precision at which the point of signal deterioration can be detected. If set to a minimum (the number of time points in a separate expression profile), the method produces results similar to the traditional approach described by Ptitsyn et al. [10–12]. However, the number of periodically expressed genes revealed by the minimal length window may be still different due to the digital filters' improvement of the signal-to-noise ratio. Increasing the length of window *W* involves more time points and thus increases the power of the statistical tests for periodicity, but on the other hand reduces the precision at which the point of signal deterioration can be located. Using a relatively small *W* equal to four single expression profiles offers a reasonable compromise between power (equivalent to a four-times-longer time series) and specificity (plus or minus four genes out of thousands represented on a microarray).

## Results

**Figure 2:**
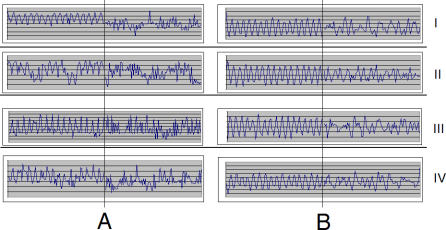
Processing of Phase Continuum by Digital Filters Concatenation of single profiles in order of descending likeliness of oscillation (same as in Figure 1) within each same-phase group results in a stream of measurements (phase continuum). Each of four phase continuums of murine liver starts with obviously circadian profiles, and each two consecutive humps correspond to one gene. The oscillation signal deteriorates towards the end of each phase continuum. Here, each plot shows only the 100 first and 100 last time points. The raw data (A) contain more noise even in the most periodic profiles (left half of each plot) and show obvious deterioration of the circadian signal at the end (right halves). After application of digital filters (B), the same part of the continuum looks nearly free of noise, and some circadian periodicity can be traced even among the least periodic genes. The latter observation is confirmed by application of a variety of statistical tests for periodicity. The figure contains screenshots of actual data scaled down to fit.

We have analyzed the microarray data from multiple microarray timeline experiments originating from independent sources and have found oscillating patterns with statistically significant circadian periodicity in as many as 99% of all genes present in the dataset (Table 1). As an alternative to the K-S test, we have also applied the Fisher's *g*-test and autocorrelation analysis in the same window *W* sliding along the phase continuum. These methods report the numbers consistent with the K-S test. The exact numbers are slightly different due to the fact that different algorithms exploit different properties of oscillation (see [12] for benchmarks of a number of periodicity tests). The digital filters improve the ability to identify oscillation pattern, but the quantitative effect is different in conjunction with the individual tests. Overall in our study, the number of oscillating genes reported approaches 100% of the total number of genes by more than one test for each particular digital filter. Likewise, each particular test reports nearly 100% oscillating genes with application of more than one different filter. The effect of the positional centering filter is noticeably different from the two others shown in Table 1. Unlike the other two filters, positional centering is an estimation based on the central value of time points separated by a complete hypothetic period (in this study, circadian), rather than smoothing of the adjacent time points. In effect, it mimics the human eye observing the heatmap in Figure 1, where all expression profiles are stacked on top of each other, while other filters mimic the human eye at it tries to follow the flow of curves in Figure 2. Detection of periodicity in a continuous stream of same-phase time points (phase continuum) has more than one challenge: not only can stochastic variation can obscure the baseline oscillation, but noisy profiles can also be misclassified and placed in the wrong phase class. The order in which single profiles are concatenated can differ slightly, depending on the preliminary estimation of oscillation likeliness by autocorrelation or a *p*-value of one of the periodicity tests [12] As a result, when periodicity is tested in a sliding window covering a number of single gene expression profiles, some windows may contain a mixture of periodic but phase-misclassified—as well as stochastically varying—fragments. The position-centering filter is more robust in evaluating the mixture of “good” and “noisy” periods within one window. On the other hand, centering may reduce the signal-to-noise ratio, which explains its lower performance in combination with Fisher's *g*-test. Experimental validation of a few selected genes involved in basic energy metabolism by qRT-PCR has confirmed the presence of an oscillating pattern in gene expression [10]. This circadian regulation of essential metabolic pathways is likely to influence the expression profile of downstream, metabolically dependent genes.

**Table 1:**
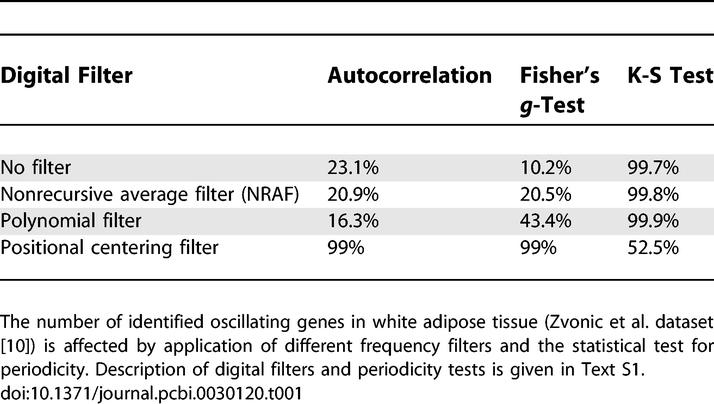
Application of Digital Filters

The number of circadian oscillating genes we report by far exceeds the expected 10%–15%, but is consistent with the pattern visible on the heatmap plot of the whole dataset grouped by phase. It is also consistent with previously reported simulation studies [10] that pointed to the absence of an identifiable nonoscillating fraction of genes in our data. We believe that the source of the discrepancy results from the intuitive, but unfounded, assumption made in the formulation of the null hypothesis applied to tests of gene expression profiles for periodicity. It is natural to suppose that gene expression profiles that fail a statistical test for periodicity are not periodic (i.e., expressed in a steady-line manner). However, there are other reasons for the test to fail: the time series acquired in a microarray or RT-PCR experiment is too short, sampled at too low a level, and/or includes a high degree of stochastic variation. Thus, failure to pass a test for periodicity does not necessarily imply a steady-line expression profile. This could simply reflect an oscillating pattern obstructed by noise. The use of RT-PCR as a standard for validation does not represent an improvement, because the number of points is no higher than that of the microarray experiment. Indeed, the low number of time points and thus low sampling rate is the main problem for identification of periodicity in gene expression profiles. Consequently, all algorithms for analysis of periodicity are at a disadvantage when applied to one gene at a time. Although our same-phase continuum approach does not eliminate the problem of low sampling rate, it increases the statistical power by grouping genes oscillating in the same phase and scaled to the same amplitude. These genes are also similar in the likelihood of their oscillating pattern because they are next to each other in the window *W*, ranked by circadian autocorrelation. As a group, these genes provide a sufficient number of time points to identify the pattern of circadian oscillation with confidence. Conversion of single gene profiles into a long continuous stream allows application of digital signal processing to reduce the noise and enhance the signal.

## Discussion

The concept that most, if not all, genes in a living cell experience oscillation in expression is not necessarily surprising. It has been previously reported that as many as more than 60% of all genes in Saccharomyces cerevisae oscillate in ultradian rhythm with a period of ~300 min [13]. In an earlier work, Klevecz et al. [14] have also demonstrated a genome-wide oscillation pattern with peaks of expression coinciding with respiratory, early, or late reductive phases of metabolic cycle. These studies used different experimental techniques and obtained different periods of oscillation as well as other details. However, both demonstrate the prominence of rhythmic patterns generated by the most important cellular mechanisms and affecting practically every active gene. This rhythm is postulated to be a primordial oscillator for modern organisms, while other rhythms can be derived by period-doubling. Reanalyzing the same data with our phase-continuum approach reveals oscillation in practically 100% of genes, which confirms the findings and provides an extra ground for the theory proposed by the authors. Much earlier studies by Selkov [5,6] have pointed out that basic cell metabolism is a natural oscillator in and of itself. His studies emphasized the role of oscillation in temporal compartmentalization of metabolic function in cyanobacteria. Circadian regulation of genes responsible for basic energy metabolism in mice has been reported by Panda et al. [15], although not extrapolated to gene expression in general. Our experimental RT-PCR validation of selected microarray timelines also confirmed periodic patterns in genes involved in regulation of oxidative phosphorylation, such as *PGC1α, LPL, PDK4,* and many others [10,11]. If genes regulating the main source of energy oscillate, this must impose oscillation on a large number of other genes, if not by direct transcription regulation, then simply by variation of energy balance. This contrasts with the relatively small number (only a few dozen to a few hundred) of circadian genes that have been reported in the other studies [15,16]. On the other hand, if temporal compartmentalization is important for oxidative phosphorylation in yeast and makes such a massive impact on the expression pattern of the absolute majority of other genes, it is reasonable to extrapolate that the same organizing principle operates in higher eukaryotes, mice and humans included, whose metabolism is also dependent on oxidative phosphorylation. Unfortunately, with the available sampling rate (one time point in 4 h) and longer periods we cannot make a decisive statement regarding gene expression oscillations in mammals as has been concluded based on the respiratory oscillation in yeast. Nevertheless, we can hypothesize that in mammals, respiratory oscillation is coordinated with the circadian rhythm observed in the vast majority of genes. Our analyses show that there is no difference in the prominence of the metabolically driven oscillation in yeast and the circadian oscillation in multiple murine tissues. Genes involved in oxidative phosphorylation and driving the ultradian oscillation in yeast have homologous counterparts in mammals, also oscillating, but synchronized with the circadian rhythm of daily activity. Among other data we have analyzed three murine liver sets obtained and published independently by Panda et al. [15], Storch et al., [16], and Zvonic et al. [10]. These data were collected in similarly designed experiments, differing with respect to the lighting regime during the sample collection period. While the Zvonic et al. data was collected in a continuous 12/12 h light/dark period, the Panda et al. data were collected in complete darkness, and the Storch et al. data were collected in constant dim light. Comparison of gene oscillation patterns indicates that neither complete exclusion nor leveling the oscillation of a major entraining factor (photic stimulation) eliminates oscillation, but does modulate the outcome. In contrast to the normal function represented in the Zvonic et al. dataset, two other murine liver sets show higher fluctuation in amplitude between periods. Phase classes in the Zvonic et al. data have approximately equal numbers of genes, while the Panda et al. and Storch et al. datasets have different, unequal numbers of genes in phase classes. This observation supports the conclusion that the principal function of mammalian circadian molecular clock is to maintain temporary timekeeping, adjustment, and synchronization between metabolic rhythms and environmental factors.

**Figure 3:**
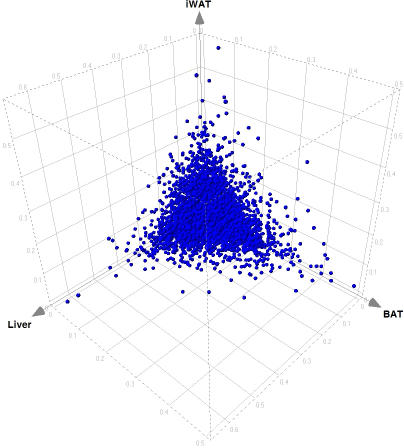
Amplitude of Oscillation Can Be Tissue-Specific Here, each point represents a gene, and axes are amplitudes of oscillation in murine liver and white and brown adipose tissues, respectively. For each gene, the amplitude is divided by the mean value (i.e., presented in a relative form independent from the overall level of expression in particular tissue). Units on the axis are fold change relative to the mean expression level through 12 time points of two complete circadian periods.

The rhythmicity of gene expression itself is important for understanding and modeling of the interaction and regulation of gene expression in the context of biological pathways. Each interaction in a biological pathway takes place under constantly changing circumstances throughout the day. Oscillation of interacting genes has to be synchronized in order to maintain specific concentrations of gene products and their metabolic substrates at a particular time. Synchronization of oscillation among cells is an essential property of each tissue, or at least of each tissue we studied. We would not be able to observe the rhythmic pattern if it was not synchronized through the cell interaction on the tissue level. However, it is likely that different mechanisms are involved in the coordination of gene expression between tissues on the organism level. Our studies indicate that while thousands of expressed genes oscillate, the phase of oscillation is highly tissue-specific (Figure S2). Relatively few genes are found to be oscillating in the same phase, even between such similar tissues as white and brown fat. In contrast, the amplitude of the oscillation is less specific. Relative fold change within a circadian period does not vary significantly between tissues for most genes (Figure 3). However, some genes show a considerable difference in amplitude between tissues. Remarkably, the amplitude of core circadian genes does not stand out from the majority of expressed genes and shows no significant variation among the tissues we studied.

**Figure 4:**
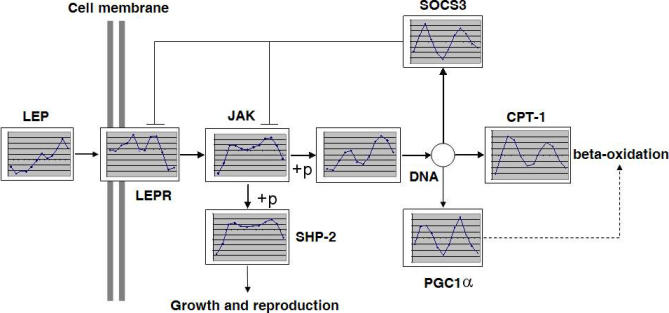
Diurnal Expression Pattern in Leptin-Signaling Pathway in Murine Liver Leptin is expressed in liver at low levels and with an unclear circadian pattern, unlike the leptin expression in adipose tissues (unpublished data). Other components of the leptin-signaling pathways in liver are expressed in a diurnal oscillating pattern. This oscillation has to be taken into account when modeling signal transduction in liver. The plot areas contain actual screenshots of circadian profiles, scaled down to fit.

If a perturbation is introduced into this oscillating system, its effect depends on the circadian time- and wave-guiding properties of the biological pathways. To illustrate this point we have superimposed the cartoon of the leptin-signaling pathway (fragment of the KEGG hsa04920 pathway) onto the expression profiles of individual genes in the liver (Figure 4). Leptin itself is hardly expressed in the liver, and thus its profile shows little evidence of periodicity. However, in adipose tissue, leptin expression is periodic (picture not shown). All other components of the pathway show evident periodicity. The timing of the incoming signal (a surge in leptin concentration) is very important, because all components of the signal transduction chain are expressed at different levels throughout the day.

At present, a researcher in systems biology has a choice of public or private databases of biological pathways and interaction networks. However, none of the available databases contains the information about oscillation of expression level for the components of biological pathways (similar to those presented in Figure 4). The biological pathways and interaction networks are typically presented in a way similar to the graphic depiction of direct current electric circuits. It is assumed that up- or downregulation of a particular component of a biological pathway causes changes in gene expression of downstream elements of the same pathway in a way similar to a direct current electric circuit. We believe that this presentation does not reflect the complex nature of gene expression and omits an important property of biological systems. Biological pathways and interaction networks should be viewed and modeled in a way more similar to the alternating current circuits, in which phase and amplitude are important characteristics of function of each component. Timing is important for understanding the effect of each biological signal as well as the waves spreading through the system perturbed by signal. Like in alternating current circuits, the likely primary source of oscillation is the “energy source” of a cell, the respiratory cycle. The circadian molecular clock based on negative feedback plays the important role of temporary timekeeping, synchronizing oscillation in gene expression levels with the major environmental factors such as daylight. The commonality of oscillation in gene expression reported in this paper suggests that oscillation is a natural and important feature of all or nearly all biological pathways. Genes that display daily variation in their expression level may or may not be linked directly to the circadian molecular clock. Regardless of mechanism, the oscillation of these genes and their encoded protein products will affect all elements of the system as a whole. Hence, the same “alternating current” principle could be applied in modeling all biological processes in mammals and, possibly, in other organisms.

## Supporting Information

Figure S1 The Algorithm of Phase Continuum Analysis of Periodicity in a Large Number of Short Gene Expression Profiles (51 KB JPG)

Figure S2 Phase of Oscillation Is Highly Tissue-Specific Four Venn diagrams present the co-occurrence of genes oscillating in the same phase among three tissues. For example, only 268 genes are found oscillating in the same phase among murine liver and brown and white adipose tissues.
(29 KB JPG)

Text S1 Supplementary Methods (131 KB DOC)

## Abbreviations

K-S: Kolmogorov-Smirnov

## References

[pcbi-0030120-b001] Albrecht U, Eichele G (2003). The mammalian circadian clock. Curr Opin Genet Dev.

[pcbi-0030120-b002] Dunlap JC, Loros JJ, Liu Y, Crosthwaite SK (1999). Eukaryotic circadian systems: Cycles in common. Genes Cells.

[pcbi-0030120-b003] Halberg F, Cornelissen G, Wang Z, Wan C, Ulmer W (2003). Chronomics: Circadian and circaseptan timing of radiotherapy, drugs, calories, perhaps nutriceuticals and beyond. J Exp Ther Oncol.

[pcbi-0030120-b004] Refinetti R (2005). Time for sex: Nycthemeral distribution of human sexual behavior. J Circadian Rhythms.

[pcbi-0030120-b005] Selkov E (1968). On the mechanism of single-frequency self-oscillations in glycolysis. A simple kinetic model. Eur J Biochem.

[pcbi-0030120-b006] Selkov E (1975). Stabilization of energy charge, generation of oscillation and multiple steady states in energy metabolism as a result of purely stoichiometric regulation. Eur J Biochem.

[pcbi-0030120-b007] Gonze D, Halloy J, Goldbeter A (2002). Robustness of circadian rhythms with respect to molecular noise. Proc Natl Acad Sci U S A.

[pcbi-0030120-b008] Ahdesmaki M, Lahdesmaki H, Pearson R, Huttunen H, Yli-Harja O (2005). Robust detection of periodic time series measured from biological systems. BMC Bioinformatics.

[pcbi-0030120-b009] Wichert S, Fokianos K, Strimmer K (2004). Identifying periodically expressed transcripts in microarray time series data. Bioinformatics.

[pcbi-0030120-b010] Zvonic S, Ptitsyn AA, Conrad SA, Scott LK, Floyd ZE (2006). Characterization of peripheral circadian clocks in adipose tissues. Diabetes.

[pcbi-0030120-b011] Ptitsyn AA, Zvonic S, Conrad SA, Scott LK, Mynatt RL (2006). Circadian clocks are resounding in peripheral tissues. PLoS Comput Biol.

[pcbi-0030120-b012] Ptitsyn AA, Zvonic S, Gimble JM (2006). Permutation test for periodicity in short time series data. BMC Bioinformatics.

[pcbi-0030120-b013] Tu BP, Kudlicki A, Rowicka M, McKnight SL (2005). Logic of the yeast metabolic cycle: Temporal compartmentalization of cellular processes. Science.

[pcbi-0030120-b014] Klevecz RR, Bolen J, Forrest G, Murray DB (2004). A genomewide oscillation in transcription gates DNA replication and cell cycle. Proc Natl Acad Sci U S A.

[pcbi-0030120-b015] Panda S, Antoch MP, Miller BH, Su AI, Schook AB (2002). Coordinated transcription of key pathways in the mouse by the circadian clock. Cell.

[pcbi-0030120-b016] Storch KF, Lipan O, Leykin I, Viswanathan N, Davis FC (2002). Extensive and divergent circadian gene expression in liver and heart. Nature.

