# Supplementary material for: Digital Signal Processing Reveals Circadian Baseline Oscillation in Majority of Mammalian Genes
Source: PLoS Comput Biol. 2007 Jun 15;3(6):e120. doi: 10.1371/journal.pcbi.0030120 (PMC1892608; doi:10.1371/journal.pcbi.0030120)
Supplement: Supplementary file 1 [file pcbi.0030120.sd001.doc]

# Supplementary Methods

## Time series data sets.

**Pennington data set.** We have completed independent circadian studies in AKR/J mice acclimated to a 12 hr light: 12 hr dark cycle, harvesting sets of 3-5 mice at 4 hr intervals in duplicates over a 24 hr period [1]. Total RNA samples from inguinal (iWAT) white adipose tissue, brown adipose tissue (BAT), and liver have been assayed by Affymetrix microarrays. A few genes have been selected for validation with RT-PCR for the expression profile of representative circadian rhythm genes in all 3 tissues. The transcriptomic data set contained over 22,000 gene expression profiles for each of 3 different tissues. In the current study, we have used only the murine liver data. Since each time point was sampled twice, the following Fourier transform for each profile can be re-arranged into a short time series that represents two complete circadian cycles. Profiles have been smoothened by a 3^rd^ degree polynomial procedure and median-subtracted. For better compatibility, the same smoothing and median subtraction procedure has been applied to all other data sets.

**Affymetrix oligonucleotide microarray gene expression analysis (Pennington Data Set)**. RNA integrity was assessed with Agilent 2100 Bioanalyzer (Agilent Technologies, Palo Alto, CA). Double-stranded cDNA was synthesized from ~9 μg total RNA using a Superscript cDNA Synthesis kit (Invitrogen, Carlsbad, CA) in combination with a T7-(dT)24 primer. Biotinylated cRNA was transcribed in vitro using the GeneChip IVT Labeling kit (Affymetrix, Santa Clara, CA) and purified using the GeneChip Sample Cleanup Module. Ten micrograms of purified cRNA was fragmented by incubation in fragmentation buffer (200 mmol/l Tris-acetate, pH 8.1, 500 mmol/l potassium acetate, and 150 mmol/l magnesium acetate) at 94°C for 35 min and chilled on ice. Fragmented biotin-labeled cRNA (6.5 μg) was hybridized to the Mouse Genome 430A 2.0 Array (Affymetrix), interrogating >14,000 substantiated mouse genes. Arrays were incubated for 16 h at 45°C with constant rotation (60 rpm), washed, and stained for 10 min at 25°C with 10 μg/ml streptavidin-R phycoerythrin (Vector Laboratories, Burlingame, CA) followed by 3 μg/ml biotinylated goat antistreptavidin antibody (Vector Laboratories) for 10 min at 25°C. Arrays were stained once again with streptavidin-R phycoerythrin for 10 min at 25°C, washed, and scanned using a GeneChip Scanner 3000. Pixel intensities were measured, expression signals were analyzed, and features were extracted using the commercial software package GeneChip Operating Software v.1.2 (Affymetrix). Data mining and statistical analyses were performed with Data Mining Tool v.3.0 (Affymetrix) algorithms. Arrays were globally scaled to a target intensity value of 2,500 to compare individual experiments. The absolute call (present, marginal, and absent) of each gene expression in each sample and the direction of change and fold change of gene expressions between samples were identified using the above-mentioned software.

**GNF data set**. This data set was provided courtesy of Dr. Hogenesch [2] and contains microarray expression profiles of nearly 10,000 genes in murine liver and hypothalamus, measured at 2hr intervals over a 48hr period. Our analysis used only the liver subset.

**Harvard data set**. This data set, provided courtesy of Dr. Storch [3], was collected from murine heart and liver; only the liver data set was used in our study. Each data set has 12 time points collected at even intervals of 4 hours over a period of 48 hours. The experimental design is similar to our own based on number of time points and the period of observation.

**Yeast data sets**. We have studies two independent data sets. One has been kindly provided by Dr. Tu and Dr. McKnight [4]. The data contains microarray expression time series of 36 samples collected at intervals of ~25 *min*. It has been reported that the whole time series covers approximately three periods of 300 *min* each and the majority of expressed genes follow this oscillating pattern. The other data set has been downloaded from the GEO database, submitted by Klevecz et al. [5]. This data contains 32 time points over three consecutive periods taken every ~4 min.

## Digital Signal Processing

**Spectral Analysis**. For purposes of spectral analysis, consider a series of microarray expression values for gene *x* with *N* samples of the form

$Y=x_{0},x_{1},x_{2},\text{.}\text{.}\text{.}x_{N-1}$.

This series can be converted from time-domain, where each variable represents a measurement in time to a frequency domain using Discrete Fourier Transform (DFT) algorithm. Frequency domain representation of the series of experiments is also known as periodogram, which can be denoted by *I*(ω) :

$I(\omega)=\frac{1}{N}\left| \sum_{t=0}^{N-1} x_{t}e^{\left( -i\omega t \right)} \right|^{2},\omega\in\left[ 0,\pi\right]$.

If a time series has a significant sinusoidal component with frequency ω$\in$[0, *π*], then the periodogram exhibits a peak at that frequency with a high probability. Conversely, if the time series is a purely random process (a.k.a “white noise”), then the plot of the periodogram against the Fourier frequencies approaches a straight line [6].

**Fisher’s g-test**. The significance of the observed periodicity can be estimated by Fisher *g*-statistics, as recently recommended in [7]. Fisher derived an exact test of the maximum periodogram coordinate by introducing the *g*-statistic

$$g=\frac{\text{max}_{k}I\left( \omega_{k} \right)}{\sum I\left( \omega_{k} \right)},$$

where $I\left( \omega_{k} \right)$ is a *k-*th peak of the periodogram. Large values of g indicate a non-random periodicity. We calculate the *p*-value of the test under the null hypothesis with the exact distribution of *g* using the following formula:

$$P\left( g>x \right)=\sum_{p=1}^{1x} \left[ \left( -1 \right)^{p}\frac{n!}{p!\left( n-p \right)!}\left( 1-\text{px} \right)^{n-1} \right],$$

where *n* = [*N/*2] and *p* is the largest integer less than 1*/x*.

This algorithm closely follows the guidelines recommended for analysis of periodicities in time-series microarray data [7] with the exception that we applied a locally developed C++ code instead of R scripts.

**Autocorrelation**. For a given a discrete time series $Y=x_{0},x_{1},x_{2},\text{.}\text{.}\text{.}x_{N-1}$ the autocorrelation is simply the correlation of the expression profile against itself with a frame shift of *k* data points (where $0\leq k\leq N-1$, often referred as the lag). For the time shift *f*, defined as $f=i+k$ if *i+k<N* and $f=i+k-N$otherwise

$$R(f)=\frac{\sum\left( x_{i}-\bar{x} \right)\left( x_{f}-\bar{x} \right)}{\sum\left( x_{i}-\bar{x} \right)^{2}}$$

For each time series we calculate the maximum positive *R(f)* among all possible phase shifts *f* and use tabulates 0.05 significance cutoff values for correlation coefficient. Time series that shows significant autocorrelation *R(f)* with the lag *f* corresponding to one day are considered circadially expressed.

**Permutated time (Pt-test)**. Consider a time series $Y=x_{0},x_{1},x_{2},\text{.}\text{.}\text{.}x_{N-1}$, in which technical variation may approach or exceed the amplitude of periodic expression The periodogram of the original time series *IY(ω)* contains a significant peak corresponding to a particular frequency (for example, circadian). Let *YR* be a random permutation of the time series *Y.* Its corresponding periodogram is *IR(ω)*. After DFT a periodogram *IR(ω)* would represent only the peaks occurring by chance. However it will miss the true periodic frequencies unless permutations happen to preserve the period, for example if the rank of each point *x* in permutated series *YR* is equal $x_{Y}\pm n*p$where *n* is a natural number and *p* is a period corresponding to a significant peak in *IY(ω)*. To avoid random re-institution of periodicity we generate *YR* by multiple shuffling of randomly selected time points $x_{n}\Leftrightarrow x_{m}$, where $\left| n-m \right|\neq p$, i.e. each shuffle is swaps time points from different phase. For each gene expression profile we generate two series of *min(n!,100)* random permutations. Each permutated series *YR* is transformed to the frequency domain and a single peak of the periodogram *IR(ω)* is stored. The p-value for the null-hypothesis of random nature of a particular peak of periodogram can be estimated by comparing the stored *IR(ω)* values to the observed *I(ω)*:

$p=\frac{N_{\text{IR}(\omega)\geq\text{IY}(\omega)}}{\text{min}\left( n\text{!,}\text{100} \right)}$.

High *p*-value exceeding the threshold, for example 0.05, means that at least 5 out of 100 random permutations of time series produce a periodogram with the same or higher peak, corresponding to a given periodicity. Low *p*-values indicate a significant difference between periodogram *IR(ω)* preserving circadian periodicity and randomly permutated periodogram *IY(ω)* with the same level of technical variation. This difference leads to rejection of the null-hypothesis of purely random nature of variation in the original time series *Y*.

**Polynomial Filter**. This digital filter is based on a procedure fitting a smooth line into an original time series$Y=x_{0},x_{1},x_{2},\text{.}\text{.}\text{.}x_{N-1}$. We use 3^rd^ degree seven-point Savitzky-Golay algorithm [8].

**Non-recursive Average Filter**. For a time series $Y=x_{0},x_{1},x_{2},\text{.}\text{.}\text{.}x_{N-1}$ the output of NRAF digital filter is $Y^{'}=x_{0}^{'},x_{1}^{'},x_{2}^{'},\text{.}\text{.}\text{.}x_{N-1}^{'}$ where$x_{i}^{'}=\frac{1}{3}(x_{i-1}+x_{i}+x_{i+1})$.

**Positional Centring Filter**. For a time series $Y=x_{0},x_{1},x_{2},\text{.}\text{.}\text{.}x_{N-1}$ with possible oscillating pattern of period *p* the output of Positional Centering Filter $Y^{'}=x_{0}^{'},x_{1}^{'},x_{2}^{'},\text{.}\text{.}\text{.}x_{N-1}^{'}$ is computed as a central value (mean, median or trimmed mean) of the time point over a few adjacent periods of oscillation, for example $x_{i}^{'}=\frac{x_{i-2p}+x_{i=p}+x_{i}+x_{i+p}+x_{i+2p}}{5}$ .

Expression

Profiles

Pre-processing

Phase

Assignment

Concatenate

Profiles in each phase class

Smoothing

Low-pass

Frequency filter

Sorting by p-value

(or autocorrelation)

Test periodicity in

Window W

p<0.05?

Finish

Shift window by one

profile length

Test for periodicity

Output the number of iterations

**Supplementary Figure 1. The algorithm of phase continuum analysis of periodicity in a large number of short gene expression profiles**.

**Supplementary Figure 2. Phase of oscillation is highly tissue-specific**. Four Venn diagrams present the co-occurrence of genes oscillating in the same phase among 3 tissues. For example, only 268 genes are found oscillating in the same phase between murine liver, brown and white adipose tissues.

References

1.	Zvonic S, Ptitsyn AA, Conrad SA, Scott LK, Floyd ZE, Kilroy G, Wu X, Goh BC, Mynatt RL, Gimble JM: **Characterization of peripheral circadian clocks in adipose tissues**. *Diabetes* 2006, **55**(4):962-970.

2.	Panda S, Antoch MP, Miller BH, Su AI, Schook AB, Straume M, Schultz PG, Kay SA, Takahashi JS, Hogenesch JB: **Coordinated transcription of key pathways in the mouse by the circadian clock**. *Cell* 2002, **109**(3):307-320.

3.	Storch KF, Lipan O, Leykin I, Viswanathan N, Davis FC, Wong WH, Weitz CJ: **Extensive and divergent circadian gene expression in liver and heart**. *Nature* 2002, **417**(6884):78-83.

4.	Tu BP, Kudlicki A, Rowicka M, McKnight SL: **Logic of the yeast metabolic cycle: temporal compartmentalization of cellular processes**. *Science* 2005, **310**(5751):1152-1158.

5.	Klevecz RR, Bolen J, Forrest G, Murray DB: **A genomewide oscillation in transcription gates DNA replication and cell cycle**. *Proc Natl Acad Sci U S A* 2004, **101**(5):1200-1205.

6.	Priestley MB: **Spectral Analysis and Time Series.** In: *.* vol. , edn. : Academic Press, London.; 1981: .

7.	Wichert S, Fokianos K, Strimmer K: **Identifying periodically expressed transcripts in microarray time series data**. *Bioinformatics* 2004, **20**(1):5-20.

8.	Savitzky A, Golay, M.: **Smoothing and differentiation of data by simplified least squares procedures**. *Analytical Chemistry* 1964, **36**:1627-1639.
